# Flares and Predicting Factors of Flares in Patients with Systemic Lupus Erythematosus Associated with Different Doses and Types of COVID-19 Vaccines

**DOI:** 10.3390/vaccines12121399

**Published:** 2024-12-12

**Authors:** Worawit Louthrenoo, Punsita Tangkum, Nuntana Kasitanon, Wanitcha Gumtorntip, Poramed Winichakoon, Supparat Konsamun, Antika Wongthanee

**Affiliations:** 1 Division of Rheumatology, Department of Internal Medicine, Faculty of Medicine, Chiang Mai University, Chiang Mai 50200, Thailand; punyii83@gmail.com (P.T.); nkasitan@gmail.com (N.K.); wanitcha.ch@cmu.ac.th (W.G.); 2 Division of Infectious Diseases and Tropical Medicine, Department of Internal Medicine, Faculty of Medicine, Chiang Mai University, Chiang Mai 50200, Thailand; poramed.win@gmail.com; 3 Research Unit, Department of Internal Medicine, Faculty of Medicine, Chiang Mai University, Chiang Mai 50200, Thailand; supparat.pueng@gmail.com (S.K.); antika.wong@cmu.ac.th (A.W.)

**Keywords:** systemic lupus erythematosus, COVID-19 vaccine, disease activity, flares

## Abstract

**Objectives**: To compare disease activity and flares among different doses and types of COVID-19 vaccines in systemic lupus erythematosus (SLE) patients. **Methods:** SLE patients in a lupus cohort, who received at least one dose of a COVID-19 vaccine (inactivated virus, adenovirus-vectored, or mRNA vaccines) between March and October 2022 joined this study. The data regarding disease activity and flares after each dose were reviewed and compared. **Results:** Two hundred and one SLE patients (524 total doses) were included in this study, with 201, 199, and 124 of them receiving 1, 2, and 3 doses of a vaccine, respectively, which comprised 183, 128, and 213 doses of inactivated virus, adenovirus-vectored, and mRNA vaccines, respectively. Regardless of vaccine dose or type, there were no significant changes in SLE disease activity pre- or post-vaccination. Flares were significantly more common after the 2nd and 3rd doses than after the 1st one (20.10% and 17.74% vs. 8.96%, *p* = 0.001, and *p* = 0.010, respectively), and after inactivated virus, adenovirus-vectored and mRNA vaccinations in 11.48%, 14.84%, and 17.84% of the patients (*p* = ns), respectively. However, the incidence rate of flares/100 patient-months was not different. The majority of flares were severe, with renal flares being the most frequent. Renal and mucocutaneous involvement and high SLE disease activity prior to the 1st vaccine dose were independent factors that predicted flares. **Conclusions:** Flares after COVID-19 vaccination were not uncommon. Most of the flares were severe, mainly due to renal flares. SLE patients should have stable low disease activity prior to receiving COVID-19 vaccine in order to avoid flares.

## 1. Introduction

COVID-19 infection caused significant mortality and morbidity worldwide during the pandemic. Vaccination is the most effective strategy for reducing its severity and mortality. Due to its very rapid spread, several prequalified COVID-19 vaccines were authorized for use in emergency circumstances. These vaccines were shown to reduce morbidity and mortality from COVID-19 infection [1,2], but they were also associated with a significant number of adverse reactions [3].

The primary series of COVID-19 vaccines consists of 2 doses given a few weeks apart. After priming by the 1st dose, the 2nd dose results in a surge in immune response, increasing inflammatory cytokines and antibody production against the virus [4,5]. However, the antibody levels tend to wane or decrease over time. Therefore, the 3rd dose is typically given several months later as a booster dose. This 3rd or booster dose, either homologous or heterologous, usually enhances immunogenicity and antibody response similar to that of the 2nd dose [6,7]. In addition, the antibody response level has been observed to be higher following an mRNA vaccine compared to the adenovirus-vectored and inactivated virus vaccines [4,6].

Patients with systemic lupus erythematosus (SLE) have an increased risk of infection, not only because of corticosteroid and immunosuppressive drug treatment but also from an aberrant immune system [8]. Infection in SLE results in significant morbidity and mortality. Studies have found that SLE patients had a higher risk of COVID-19 infection and a higher rate of severe disease, hospitalization, and death than the general population [9,10]. Furthermore, studies have shown that SLE patients had a lower seroconversion rate and a lower antibody level against the SARS-CoV-2 spike protein when compared to people in general [11,12]. In addition, due to the antibody level usually waning or decreasing over time, ≥2 additional booster doses are recommended for SLE patients after completing 3 doses of the primary series [13]. It has been shown that vaccinated SLE patients had similar risks and adverse outcomes from COVID-19 infection to vaccinated people in general [10].

The effects of COVID-19 vaccines on SLE disease activity and flares have been determined by several groups [11,14,15,16,17,18,19,20,21,22,23,24,25,26,27,28,29] and a recent systemic review [12]. Interestingly, despite a notable number of flares, their incidence rate after each vaccine dose has been compared rarely, and with conflicting results [14,21,22,25]. Moreover, flares between vaccine types have never been compared or mentioned in studies of mixed types of vaccines [17,18,20,28,29].

Different antibody levels to the SARS-CoV-2 spike protein have been shown among doses and types of vaccines after COVID-19 vaccination, which might reflect differences in the underlying immunologic reaction and inflammatory cytokines production [4,5]. Whether these differences in immune response trigger SLE exacerbation or flares differently after various doses or types of COVID-19 vaccines is unknown.

Therefore, this study aimed to determine and compare flare incidence, organ flares, and flare severity among SLE patients receiving different doses and types of COVID-19 vaccines.

## 2. Materials and Methods

### 2.1. Study Design and Participants

This retrospective study was conducted at the outpatient rheumatology clinic between March and October 2022. During that period, all consecutive SLE patients in the Chiang Mai University Lupus Cohort were invited to join this study. These patients were regularly followed up at 1- to 3- month intervals, depending on their disease activity. The inclusion criteria were age ≥ 20 years and having received at least 1 dose of a COVID-19 vaccine. The exclusions were SLE patients with overlapping rheumatic diseases (such as systemic sclerosis, rheumatoid arthritis, and vasculitis syndrome, etc.) or those with a history of COVID-19 infection. SLE was diagnosed according to the 1997 American College of Rheumatology (ACR) Updating Criteria for Classification of SLE [30] or the 2012 SLE International Collaboration Clinics (SLICC) classification criteria for SLE [31]. All of the participants provided their written informed consent prior to entering the study.

### 2.2. Vaccines

Three types of COVID-19 vaccines: (1) an inactivated virus vaccine (CoronaVac [Sinovac Biotech, Beijing, China] and BBIBP-CorV or Covilo [Sinopharm, Shanghai, China]), (2) an adenovirus-vectored vaccine (AZD1222 or Covishield [AstraZeneca, Cambridge, United Kingdom]) and (3) an mRNA vaccine (BNT162b2 or Comirnaty [Pfizer-BioNTech, New York, NY, USA] and mRNA-1273 or COVID-19 Moderna [Moderna, Cambridge, MA, USA]) were available in Thailand. They were provided by the government and health organizations. The inactivated virus vaccine (CoronaVac) was first available in February 2021, followed by the other two a few months later. Individuals who wanted the COVID-19 vaccine had to make an appointment and indicate the type required via the “Moh-Prompt” application on their smartphone. Due to the limited number of vaccines and the availability of each type in each period, each individual could receive either a single- or mixed-type vaccine.

### 2.3. Data Collection

The demographics, clinical features, laboratory findings, and treatment received were collected from the available data in the medical records of the patients and used for the disease activity and flare assessment. The data were collected from 6 months prior to the 1st vaccine dose to 90 days after the last dose or study censor. If a patient had a COVID-19 infection during the study period, only the prior clinical data were used for the analysis.

The vaccination date and vaccine type were obtained from the “Moh-Prompt” application. Due to the vaccination date being different from the clinic appointment, clinical manifestations and laboratory findings from the last visit pre-vaccination were used to determine disease activity on the vaccination date. Patients with significant clinical symptoms, which required medical treatment after the vaccination, were asked to visit the clinic for clinical assessment, when disease activity and flares were determined by clinical manifestations and laboratory findings.

### 2.4. Disease Activity, Flares, and Organ Damage Measurement

SLE disease activity was determined by the modified SLE Disease Activity Index 2000 (mSLEDAI-2K) [32], due to anti-dsDNA and complements not being determined routinely at this institution. The severity of SLE disease activity was classified in accordance with Abrahamowicz et al. [33], but the mSLEDAI-2K score was used instead of the original SLEDAI-2K score. Therefore, the severity of disease activity was described as remission, mild, moderate, high, or very high when the mSLEDAI-2K score was 0, 1–5, 6–10, 11–19, or ≥20, respectively. Flares and flare severity were determined by the Safety of Estrogens in Lupus Erythematosus National Assessment (SELENA) SLE flare index (SFI) [34], using the mSLEDAI-2K score. Organ damage was determined by the SLICC/ACR Damage Index (SDI) [35].

### 2.5. Statistical Analysis

The STATA 16.0 for Windows computer software (Stata Corporation, College Station, TX, USA) was used to perform the statistical analysis. The continuous variables were presented as mean ± standard deviation (SD) or median (interquartile range, p25–p75) where appropriate. The categorical variables were described as percentages. Student’s T-test or the Mann–Whitney U test were used for comparison of the continuous variables, and the Chi-square or Fisher exact test were used for the categorical variables where appropriate. For repeatedly measured continuous and dichotomous outcomes, the Repeated Measures Mixed Model and Conditional Poisson regression were used, respectively. Cox regression was used to determine the factors associated with flares. Schoenfeld residuals were applied to test violation of the proportional hazard assumption. Logistic regression analysis was used to determine the factors associated with flares. A *p*-value of <0.05 was considered statistically significant.

### 2.6. Sample Size Calculation

Assuming that approximately 10% (with a range of 5–15%) of SLE patients in this study had a disease flare after COVID-19 vaccination, at a power of 80% and alpha error of 0.05, a minimum of 139 patients was required for participation.

## 3. Results

### 3.1. Demographic Characteristics

Two hundred and one SLE patients (95% females) participated in this study. Their mean ± SD age and SLE disease duration were 41.37 ± 12.99 and 13.86 ± 8.56 years, respectively. Hypertension, dyslipidemia, thalassemia, thyroid disease, and diabetes mellitus were common co-morbidities. None of the patients were current smokers or alcohol drinkers. The demographic characteristics of the patients studied, cumulative manifestations according to the 1997 ACR classification criteria for SLE, current organ involvement at the 1st vaccine dose, SLE disease activity from 6 months prior to the 1st vaccine dose, and current medications are shown in Table 1. Almost one-third of the patients (31.34%) had renal involvement at enrollment, with most of them having a stable renal function with a 24 h urine protein of <1.0 g or a urine protein/creatinine ratio (UPCR) of <1.0 (g protein/g creatinine), and they were in the maintenance phase of active lupus nephritis treatment. They also had low disease activity from 6 months prior to the 1st vaccine dose (median [IQR] mSLEDAI-2K: 0 [0–4]).

Overall, 201, 199, and 124 patients received 525 vaccine doses, comprising 1, 2, and 3 doses, respectively. Data on the 3rd dose were excluded from one patient who had a COVID-19 infection after the 2nd dose, which left 524 vaccine doses available for analysis. These comprised 183 (34.92%), 128 (24.43%), and 213 (40.65%) doses of inactivated virus, adenovirus-vectored and mRNA vaccines, respectively. Of these, two patients received only 1 dose of a vaccine (one of each of the inactivated virus and mRNA vaccines), and the remaining 199 and 124 patients received 2 and 3 vaccine doses, respectively. The average number of data collections during the study period, from 6 months prior to the 1st vaccine dose to the study censor, was approximately 5–6 visits/patients. Immunosuppressive drugs were discontinued 1–2 weeks before and after the 1st, 2nd, and 3rd vaccine dose in 6.67% and 7.69%, 5.21% and 7.29%, and 6.72% and 6.72% of patients, respectively.

### 3.2. SLE Disease Activity and Disease Flares According to Vaccine Doses

The changes in SLE disease activity and flares according to the vaccine doses are shown in Table 2. The mSLEDAI-2K score compared between the vaccination date and the last observation date (or study censor) showed no significant differences among the 1st, 2nd, or 3rd vaccine doses. However, the mSLEDAI-2K score on the last observation date tended to be higher after the 2nd and 3rd vaccine dose than after the 1st one, but without statistical significance.

Flares occurred after the 1st, 2nd, and 3rd doses in 8.96%, 20.10%, and 17.74% of the patients, respectively. The flares were mild to moderate in 2.99%, 11.56%, and 8.06%, respectively, and severe in 5.97%, 8.54%, and 9.68%, respectively (Table 2). Although the number of total flares was higher after the 2nd and 3rd dose than after the 1st one (*p* = 0.001 and *p* = 0.010, respectively), the incidence rate (IR) and 95% confidence interval (95% CI) of flares/100 person-months were not different (IR [95% CI] of 9.08 [4.87–13.29], 8.03 [5.70–10.35], and 5.49 [3.37–7.61], respectively). Renal, mucocutaneous, and hematological systems were common organs that flared. Flares in the mucocutaneous and hematological systems developed primarily due to new rashes or alopecia and leukopenia, whereas flares in the renal system were primarily due to a significant increase in proteinuria.

### 3.3. SLE Disease Activity and Disease Flares According to Type of Vaccine

The changes in SLE disease activity and flares according to vaccine type are shown in Table 3. The mSLEDAI-2K score compared between the vaccination date and the last observation date (or study censor) showed no significant difference between the inactivated virus, adenovirus-vectored, and mRNA vaccines. However, the mSLEDAI-2K score at the last observation was higher in relation to the mRNA vaccine compared to the adenovirus-vectored vaccine (2.15 ± 2.94 vs. 2.02 ± 2.80, *p* = 0.049).

Flares occurred after inactivated virus, adenovirus-vectored, and mRNA vaccines in 11.48%, 14.06%, and 17.84% of the patients, respectively. The flares were mild to moderate in 6.01%, 6.25%, and 8.45% of the patients, respectively, and severe in 5.46%, 7.81%, and 9.39% of the patients, respectively. Although the number of total flares seemed to be higher in relation to the adenovirus-vectored and mRNA vaccines than the inactivated virus vaccine, the IR and 95% CI of flares/100 person-months were not different among these three vaccines (IR [95% CI] of 8.88 [5.19–12.57], 6.55 [3.85–9.25], and 7.12 [4.76–9.48], respectively). The renal, mucocutaneous, and hematological systems were common organs that flared similarly to those observed according to the vaccine dose (Table 3).

### 3.4. Predicting Factors for SLE Flares

The clinical characteristics of the SLE patients with or without flares after vaccination were compared (Table 4). Those with flares were younger (37.26 years vs. 43.21 years, *p* = 0.001), and had more renal and mucocutaneous involvement at the 1st vaccine dose (48.39% vs. 23.74%, *p* = 0.001 and 27.42% vs. 5.76%, *p* <0.001, respectively), and a higher mean number of the 1997 ACR and 2012 SLICC classification criteria (5.92 vs. 5.41, *p* = 0.007 and 7.73 vs. 6.75, *p* <0.001, respectively). The use of corticosteroids, hydroxychloroquine, and immunosuppressive drugs was not different, except for the flare group that used more cyclosporine (17.74% vs. 7.19%, *p* = 0.024). The patients with flares also had higher disease activity (mSLEDAI-2K) from 6 months prior to the 1st vaccine dose (3.56 vs. 1.50, *p* < 0.001). Variables with a *p*-value < 0.2 or clinical relevance in the univariable analysis were included in the multivariable logistic regression analysis. Renal and mucocutaneous system involvement prior to the 1st vaccine dose were independent predicting factors for flares, with a hazard ratio and 95% confidence interval (HR [95% CI]) of 2.57 [1.47–4.50] and 2.45 [1.31–4.60], respectively. The mSLEDAI-2k score was also a predicting factor for flares, with an HR [95% CI] of 1.13 [1.05–1.20] when excluding renal and musculoskeletal involvement in the analysis (Table 5).

### 3.5. Effect of SLE Disease Activity on Disease Flares

To determine the effect of SLE disease activity on flares, a comparison of the rate of flares was determined between the SLE patients in clinical remission or with low disease activity (mSLEDAI-2K score = 0–5) and those with moderate or higher disease activity (mSLEDAI-2K score ≥ 6) at the time of the 1st vaccine dose, regardless of the treatment received (Table 6). The patients with moderate or higher disease activity had more flares at the 1st vaccine dose (30.43% vs. 6.18%, *p* < 0.001), but not at the 2nd or 3rd doses. This also was observed with the inactivated virus and mRNA vaccines (27.59% vs. 8.44%, *p* = 0.008, and 46.67% vs. 16.67%, *p* = 0.001, respectively), but not with the adenovirus-vectored vaccine.

## 4. Discussion

This study found that regardless of the doses and types of COVID-19 vaccines, flares were observed in 8.96–20.10% of the patients. Flares were significantly more common after the 2nd and 3rd vaccine doses than after the 1st one, and numerically higher among the mRNA and adenovirus-vectored vaccines than in the inactivated virus vaccine. However, the IR of flares/100 patient-months was not different. There were no significant changes in SLE disease activity pre- or post-vaccination. Flares commonly occurred in the renal and mucocutaneous systems. The majority of flares were severe due to renal flares being the most frequent. Renal and mucocutaneous involvement and high SLE disease activity prior to the 1st vaccine dose were independent factors that predicted flares.

Flares among SLE patients receiving COVID-19 vaccine have been reported by several groups with variable results. Among the single-type vaccines, mRNA vaccines were used widely, whereas fewer inactivated virus vaccines were utilized, with a flare incidence range of 0–20%, and 11–20%, respectively (Supplementary Table S1). A prospective study in Italy by Mormile et al. [24] showed no flares among 41 SLE patients (SLEDAI score < 6 in 56%) who received 2 doses of an mRNA vaccine, whereas a retrospective study in Japan by Yoshida et al. [23] found that 20% of 74 SLE patients had flares (SLEDAI score < 6 in 43%, 6–10 in 35%, and ≥11 in 22%). This discrepancy might be due to 22% of the Japanese patients having high to very high disease activity at the time of vaccination. Flares were observed in 20% of 72 Iranian SLE patients who received at least 1 dose of an inactivated virus vaccine [22]. The majority of flares (70%) were considered mild to moderate and involved the musculoskeletal and mucocutaneous systems [11,15,16,21,22,23,25,26]. Notably, a retrospective study in Puerto Rico by Gonzalez-Melendez et al. found that 6% of 247 SLE patients who received at least 1 dose of mRNA vaccine had flares [14] involving internal organs (mainly kidney, central nervous system, lung, and liver) that were classified usually as severe. Another cross-sectional study in China by Fan et al. [26] used web-based questionnaires and found a flare incidence of 11% in 614 SLE patients who received at least 1 dose of an inactivated vaccine, of which only 3% required escalation of treatment. Unfortunately, no details of the flares were provided.

Among the mixed types of vaccines, flare incidence was reported as 0–9% of patients (Supplementary Table S1). In addition to the diverse methodology mentioned above, the proportion of the types of vaccines received might affect flares. Interestingly, two studies from Hong Kong found a discrepancy in their results. A retrospective study by Mok et al. [19] found that flares (90% of them were mild to moderate, but almost half involved the renal system) occurred in 8% of 449 SLE patients (mostly with the disease in quiescence) who received 2 doses of an inactivated virus or mRNA vaccine. In contrast, a prospective study by So et al. [20], which covered 65 SLE patients (mean SLEDAI score at 1st vaccine dose: 2.9 ± 2.0), found no flares, and, interestingly, more patients improved numerically than deteriorated in SLEDAI-2K, anti-dsDNA levels, and proteinuria after vaccination. Another two prospective studies from Thailand [27] and Denmark [18] found that SLE patients receiving at least 2 doses of COVID-19 vaccine had no flares. The patients in both studies had rather low disease activity at the time of the 1st vaccine dose. Web-based surveys in France (the international vaccination against COVID in systemic lupus [VACOLUP] study) [28] and the United States (the COVID-19 Global Rheumatology Alliance: GRA study) [29] observed 3% and 7% of medically confirmed flares, respectively. The flare incidence in this study was comparable to those in previous reports (Supplementary Table S1).

The difference in flare incidence among the aforementioned studies might be due to their diverse design (e.g., ethnicity, methods [prospective, retrospective, cross-sectional, or web-based interview], type of vaccine [single or mixed], time of assessment [duration from 1st or last vaccine dose], disease activity measurement instrument, and definition of flares and flare instruments). Therefore, awareness of the interpretation and comparison of results between studies is essential.

This study found that the flares occurring after the 2nd and 3rd vaccine doses were significantly higher than those after the 1st one. Although flares after COVID-19 vaccination have been reported widely, few studies have mentioned flare incidence in relation to vaccine dose (mostly single-type vaccines). The incidence was higher after the 2nd dose than the 1st one, as reported by Barbhaiya et al. [21] (mNRA: 67% vs. 33%, respectively), Delkash et al. [22] (inactivated virus: 43% vs. 57%, respectively), Gonzalez-Melendez et al. [14] (mRNA: 36% vs. 64%, respectively), and Zavala-Flores et al. [25] (mRNA: 9% vs. 20%, respectively). Flares among the mixed types of vaccines were not compared or mentioned [17,19,20,28,29]. However, this study found that flare incidence was higher in relation to the mRNA vaccine than in relation to the inactivated virus vaccine, which almost reached statistical significance (*p* = 0.06) (Supplementary Table S1).

The reason for the significantly higher incidence of flares after the 2nd and 3rd vaccine doses than after the 1st one is not understood clearly. A possible explanation might be that after receiving the 1st dose of the vaccine (either a whole inactivated virus, adenovirus-vectored, or mRNA vaccine), the immune system recognizes the specific portion of the virus that is used to produce the vaccine (together with its adjuvants), which stimulates the innate and adaptive immune systems, resulting in inflammatory cytokines and antibody production against the virus. These arrays of immune response can react and trigger the inflammatory system or flares in persons susceptible to autoimmune rheumatic disease [36,37]. Cytokines and antibodies responses usually occur within 0–2 and 2–3 weeks, respectively, depending on the type of vaccine [4,5]. The surge in the immune response following the 2nd dose (usually given 3–4 weeks after the 1st one) occurs because it has been primed by the initial dose [38,39,40]. During these periods, these intense inflammatory cytokines can trigger susceptible SLE patients and cause disease flares (flares are usually assessed 4 weeks after the 1st dose or just prior to the 2nd one), and the flares could be at a higher rate after the 2nd dose (usually assessed 4–12 weeks after the 2nd dose), which corresponds with the immune response. As the mRNA vaccine was given usually as the 2nd and 3rd dose, the rather high incidence of flares among patients receiving it might be explained by this mechanism. In addition, the higher rate of flares could be explained by the antibody or immunologic response being higher in relation to the mRNA and adenovirus-vectored vaccines than in the inactivated virus vaccine [20,21,22,23,24,25,26,27]. Several cases of new-onset autoimmune disease or flares after mRNA and adenovirus-vectored vaccination have been reported, which might support this hypothesis, due to the high immune response from these two types of vaccines [36,37].

The incidence of flares after mRNA vaccination seemed to be numerically higher in relation to the inactivated virus or mixed types of vaccines (Supplementary Table S1). This study found that the incidence of flares after receiving mRNA and adenovirus-vectored vaccines was numerically higher than after the inactivated virus vaccine, but the IR of flares/100 person-months was not different among the types of vaccines. This IR also was not different among the vaccine doses. This finding could be attributed to the varying time intervals between the vaccination and assessment dates for each dose or type of vaccine, as the intervals were longer for the 2nd and 3rd vaccine doses than the 1st one and longer in the mRNA and adenovirus-vectored vaccines than in the inactivated virus vaccine. Unfortunately, previous studies on disease flares in SLE patients after COVID-19 vaccination did not report them in terms of the IR (Supplementary Table S1). Therefore, direct comparison of flares between these studies is not possible.

This study found that slightly more than half of the patients had severe flares, regardless of vaccine dose or type (Table 2 and Table 3). This result was in contrast to several other studies, in that most of their patients had mild to moderate flares, with only a small proportion having severe ones [11,15,21,22,23,25,28]. The high incidence of severe flares was mainly due to renal ones. The high incidence of renal flares in this study might be due to 76% of the patients having a history of renal involvement that was still active in 31% of them at time of the 1st vaccine dose (proteinuria > 0.5 g/day or UPCR > 0.5). Many of these patients had new-onset or increasing proteinuria (>0.5 g/day or UPCR of >0.5) following vaccination and required an increase in medicine dosage or new medications added, thus fulfilling the definition of severe flares [34]. This finding is similar to those reported by Mok et al. and Gerosa et al. [17,19] who also found that renal flares were common in their patients.

Despite the notable number of flares after vaccination in several studies, most of them, including this study, found no significant changes in disease activity between the pre-vaccination and assessment periods [11,14,17,18,20,23,24], except for one from Japan that found significantly increased SLE disease activity after the 2nd dose [15]. This might be explained by the small proportion of patients with flares (approximately 10% or less), which were usually mild to moderate; therefore, the overall mean scores at the post-vaccination period did not change significantly when compared to the pre-vaccination values. In addition, when flares occurred in the same organ that had scores prior to vaccination (e.g., increasing rashes, alopecia, number of arthritic joints, proteinuria, etc.) the score at the post-vaccination period was the same. However, in these cases, flares were captured by the addition of new therapy or an increasing dosage of medication according to the SELENA flare index [34] or other definitions of flares.

Most studies of flares in patients receiving mixed types of vaccines did not compare changes in disease activity in relation to the vaccine types received (Supplementary Table S1) [17,18,19,20,27,28,29]. A study in Hong Kong by So et al. found no significant changes in disease activity between patients receiving the mRNA vaccine and those having the inactivated virus one [20]. Similarly, this study found no difference in disease activity among the patients receiving inactivated virus, adenovirus-vectored, or mRNA vaccines.

Despite flares being reported in several studies, factors associated with patients with and without them have been reported rarely (Supplementary Table S1) [14,15,19,25,28]. A retrospective study of the single-type vaccine in Puerto Rico by Gonzalez-Melendez et al. found that a higher baseline SLEDAI score and a higher proportion of photosensitivity, mouth ulcers, anti-Ro antibodies, past exposure to pulse methylprednisolone therapy, and current corticosteroid use were observed more in patients with flares [14]. A prospective study in Japan by Kikuchi et al. found that a higher SLEDAI score, a higher anti-dsDNA antibody level, a higher proportion of rash, and use of azathioprine prior to the 1st vaccine dose were observed more in patients with flares, but only the SLEDAI score and anti-dsDNA antibody titers were factors also associated with them [15]. Another prospective study in Peru by Zavala-Flores et al. found that renal involvement and hydroxychloroquine use decreased the risk of flares, but azathioprine used prior to immunization increased it [25]. Among the mixed types of vaccines studied, a retrospective study by Mok et al. in Hong Kong found that younger age, glucocorticoid treatment received in the past 3 months, and a history of discoid lesions, arthritis, positive anti-Sm/nRNP antibodies, and current active lupus serology more likely contributed to flares, but active lupus serology prior to vaccination, a history of arthritis, and discoid lesions were factors associated with flares [19]. The international vaccination against COVID in systemic lupus (VACOLUP) web-based survey found that SLE flares in the past year were associated with flares post-vaccination [28]. This study found that younger age at onset, current renal and mucocutaneous involvement, a high mSLEDAI-2K score, and a high mean number of cumulative 1997 ACR or 2012 SLICC criteria more likely contributed to flares. Renal and mucocutaneous involvement or mSLEDAI-2K scores were predicting factors of flares. Flares also were more common in patients with an mSLEDAI-2K ≥6 prior to the 1st vaccine dose. These findings are in line with previous reports in which flares usually occurred in patients with high baseline disease activity and active serology [14,15,19]. Therefore, to minimize the chance of flares, SLE patients should be in stable remission or a state of low disease activity prior to receiving a COVID-19 vaccine.

It should be noted that the adenovirus-vectored vaccine was used in only 1.6% of European SLE patients in Italy [17] and 6.5% in Denmark [18], whereas a high proportion was used in Thailand [27], including this study. SLE patients are clearly at risk of thrombosis from several mechanisms [41]. The low prevalence in European SLE patients might be related to the fear of thrombosis, with an increasing risk shown after adenovirus-vectored vaccination [42,43]. The rather high prevalence of its use in Thailand might be due to unavailability or shortage of the mRNA vaccine during that period, and those wanting to receive this vaccine had to register for either inactivated virus or adenovirus-vectored vaccines instead. Luckily, no thrombotic episodes were reported among Thai SLE patients receiving the adenovirus-vectored vaccine.

This study has several limitations. Due to its retrospective nature, some clinical data might have been missing, possibly making the disease assessment inaccurate. However, all of the patients in this study were in the Lupus Cohort and followed up regularly at 1- to 3-month intervals. Thus, the possibility of missing clinical and laboratory data is minimal. Also, the vaccination and assessment dates were different, with clinical manifestations and laboratory findings at the last visit pre-vaccination used to determine disease activity on the vaccination date. Therefore, disease activity and flares might be inaccurate in cases of minor flares being resolved without medical treatment prior to the assessment date. However, overall flares occurred in only a small proportion of patients; therefore, those who were missed should be minimal. Use of the mSLEDAI-2K instrument (excluding anti-dsDNA and complements) in SLE disease activity or flare assessments might show disease activity and flare incidence to be lower than it should be. Thus, the results from this study might be unable to be compared directly with those using the original SLEDAI-2K. However, the mSLEDAI-2K instrument has shown very good correlation with the original instrument [32,44]. The small number of patients in each subgroup, according to the dose and type of vaccine, might impact the power in the statistical analysis. However, this sample size was comparable or even larger than many single-center studies that have been reported previously (Supplementary Table S1). In addition, this study followed up the patients only 90 days after the last vaccine dose; therefore, it could capture only flares that developed early post-vaccination. Longer duration of follow-up would be more helpful in assessing the impact of COVID-19 vaccination on the clinical course of SLE patients. Furthermore, due to the characteristics of flares not being determined prior to the COVID-19 era, it is unknown whether flares that occurred after COVID-19 vaccination were taking a natural course in SLE or were related directly to COVID-19 vaccines. Lastly, the mixed types of vaccines contributing to flares in this study could not be excluded totally.

However, this study has some strengths. All of the patients were followed up regularly at 1- to 3-month intervals. Therefore, the clinical manifestations and laboratory parameters were collected properly, thus ensuring their reliable use in the assessment. The disease activity was captured from 6 months prior to the 1st vaccine dose to determine SLE disease activity prior to vaccination. Lastly, this study compared disease activity and flares among different types of vaccines, whereas most previous reports did not, which could be another strength.

## 5. Conclusions

This study found no significant changes in SLE disease activity between pre- and post-vaccination assessment, regardless of the vaccine dose or type. Although flares were not uncommon, their incidence rate was no different among them. Most of the flares were severe, mainly due to the renal flares. Renal and mucocutaneous involvement or high SLE disease activity at the time of the 1st vaccine dose were independent factors predicting flares. SLE patients should have stable low disease activity prior to receiving COVID-19 vaccines in order to avoid flares.

## Figures and Tables

**Table 1:** Baseline characteristics of the patients studied.

Baseline Characteristics		Total (N = 201)		
Female		191 (95.02)		
Age in years		41.37 ± 12.99		
SLE disease duration in years		13.86 ± 8.56, 7.02 [12.91–19.49]		
**Co** **-morbidities ** *				
Hypertension		65 (32.34)		
Dyslipidemia		65 (32.34)		
Thyroid disease		15 (7.46)		
Diabetes mellitus		13 (6.47)		
Previous malignancy		4 (1.99)		
Hepatitis B or C infection		4 (1.99)		
Others ^#^		4 (1.99)		
**Cumulative 1997 ACR manifestations**				
Malar rash		119 (59.20)		
Oral/nasal ulcers		58 (28.86)		
Photosensitivity		33 (16.42)		
Discoid rash		62 (30.85)		
Arthritis		124 (61.69)		
Serositis		27 (13.43)		
Neurological disorder		22 (10.95)		
Renal involvement		153 (76.12)		
Hematologic disorder		154 (76.62)		
ANA positive		201 (100.00)		
**Immunologic, n/N (%)**				
Anti-dsDNA antibody		150/184 (81.52)		
Anti-Sm antibody		13/30 (43.33)		
Anti-phospholipid antibodies		15/103 (14.56)		
Number of 1997 ACR classification criteria		5.57 ± 1.24, 5 [5–6]		
Number of 2012 SLICC classification criteria		7.05 ± 1.85, 7 [6–8]		
SDI score		0.91 ± 1.22, 0 [0–2]		
**Current active organ involvement** **^†^**				
Renal system		63 (31.34)		
Mucocutaneous system		25 (12.44)		
Musculoskeletal system		5 (2.49)		
Hematological system		5 (2.49)		
Gastrointestinal system		2 (1.00)		
Neurological system (central and peripheral)		1 (0.50)		
**mSLEDAI** **-2K score**				
6 months prior to 1st vaccine dose		2.32 ± 3.18, 0 [0–4]		
3 months prior to 1st vaccine dose		2.18 ± 3.33, 0 [0–4]		
1st vaccine dose (D1)		2.13 ± 3.31, 0 [0–4]		
**Baseline medications** **^†^**				
Prednisolone		174 (86.57)		
Dose in mg/day		7.45 ± 6.84, 5 [5–10]		
Hydroxychloroquine		67 (33.33)		
Dose in mg/day		150.07 ± 69.33, 200 [100–200]		
Immunosuppressive drugs		106 (52.74)		
Mycophenolate mofetil		81 (40.30)		
Dose in mg/day		1437.96 ± 702.19, 2000 [1000–2000]		
Cyclosporine		21 (10.45)		
Dose in mg/day		133.33 ± 48.30, 100 [100–200]		
Azathioprine		14 (6.97)		
Dose in mg/day		51.79 ± 22.92 50 [50–50]		
Methotrexate		6 (2.99)		
Dose in mg/week		10.83 ± 3.76, 10 [10–15]		
Cyclophosphamide		6 (2.99)		
Dose in mg/month		1033.33 ± 476.10, 1000 [1000–1500]		
Tacrolimus		2 (1.00)		
Dose in mg/day		1.25 ± 1.06, 1.25 [0.5–2.0]		
**Type of vaccine**	**1st dose** **(201 patients)**		**2nd dose** **(199 patients)**	**3rd dose** **(124 patients)**
Inactivated vaccine (183 doses)	116 (57.71)		67 (33.67)	0
Adenovirus-vectored vaccine (128 doses)	38 (18.91)		66 (33.17)	24 (19.35)
mRNA vaccine (213 doses)	47 (23.38)		66 (33.17)	100 (80.64)

Data are expressed as mean ± SD, median [p25–p75], or n (%), n/N = number of positive tests/number tested. * = one patient might have more than one co-morbid disease, ^#^ = major depression in two patients, cerebrovascular disease in two patients, end-stage renal disease in one patient and congestive heart failure in one patient, ^†^ = at time of the 1st vaccine dose. ACR = American College of Rheumatology, ANA = anti-nuclear antibody, anti-dsDNA = anti-double stranded DNA antibody, anti-phospholipid antibodies = positive in any anti-cardiolipin antibodies or lupus anti-coagulant or beta-2-glycoprotein1 antibodies, anti-Sm = anti-Smith antibody, mSLEDAI-2K = modified Systemic Lupus Erythematosus Disease Activity Index-2000, SDI = Systemic Lupus International Collaborating Clinics/American College of Rheumatology Damage Index, SLICC = Systemic Lupus Erythematosus International Collaboration Clinics.

**Table 2:** Mean mSLEDAI-2K score, flares, and organ flares according to vaccine dose.

	Dose of Vaccine		
	1st Dose (N = 201)	2nd Dose (N = 199)	3rd Dose (N = 124)
Duration of observation after vaccination (in days)	30.69 ± 25.59, 22 [20–31]	77.81 ± 43.33, 76 [44–106]	103.83 ± 39.75, 101 [74–131]
**mSLEDAI-2K score**			
Vaccination date	2.13 ± 3.31, 0 [0–4]	2.02 ± 3.14, 0 [0–4]	2.02 ± 2.98, 0 [0–4]
Assessment date *	2.06 ± 3.14, 0 [0–4]	2.16 ± 3.19, 0 [0–4]	2.25 ± 2.85, 0 [0–4]
**Flares (events)**			
Mild to mod. flares	6 (2.99)	23 (11.56)	10 (8.06)
Severe flare	12 (5.97)	17 (8.54)	12 (9.68)
Total flares (mild to mod. + severe)	18 (8.96)	40 (20.10) [*p* = 0.001] ^a^	22 (17.74) [*p* = 0.010] ^b^
**Organ flares**			
Mucocutaneous system (alopecia, skin rash, vasculitis rash)	3 (1.49)	14 (7.04)	6 (4.84)
Hematological system (AIHA, leukopenia, thrombocytopenia)	2 (1.00)	7 (3.52)	1 (0.81)
Musculoskeletal system (myositis, arthritis)	0	3 (1.51)	1 (0.81)
Renal system	11 (5.47)	14 (7.04)	12 (9.68)
Nervous system (CNS and peripheral)	1 (0.50)	1 (0.50)	0
Gastrointestinal system	0	1 (0.50)	0
Constitutional symptoms	1 (0.50)	0	2 (1.61)
**IR (95% CI) of flares (events/100 person-months**)	9.08 (4.87–13.29)	8.03 (5.70–10.35)	5.49 (3.37–7.61)

Data are expressed as mean ± SD, median [p25–p75], or n (%); IR (95% CI) = incidence rate (95% confidence interval). * = last observation day prior to the next vaccine dose or study censor. Only significant *p*-values are shown. ^a^ = *p*-value of total flares between the 1st and 2nd dose, ^b^ = *p*-value of total flares between the 1st and 3rd dose. AIHA = autoimmune hemolytic anemia, CNS = central nervous system, mSLEDAI-2K = modified Systemic Lupus Erythematosus Disease Activity Index-2000. mSLEDAI-2K: analyzed by repeated mixed models; flares: analyzed by Poisson regression; incidence rate: analyzed by Poisson regression.

**Table 3:** Mean mSLEDAI-2K score, flares, and organ flares according to type of vaccine.

	Type of Vaccine		
	Inactivated Virus (N = 183)	Adenovirus-Vectored (N = 128)	mRNA (N = 213)
Duration of observation after vaccination (in days)	41.23 ± 37.55, 24 [21–57]	70.59 ± 43.62, 74 [30–100]	84.26 ± 47.02, 85 [47–116]
**mSLEDAI-2K score**			
Vaccination date	2.38 ± 3.66, 0 [0–4]	2.01 ± 2.91, 0 [0–4]	1.83 ± 2.82, 0 [0–4]
Assessment date *	2.23 ± 3.45, 0 [0–4]	2.02 ± 2.80, 0 [0–4]	2.15 ± 2.94, 0 [0–4] (*p* = 0.049) ^a^
**Flares (events)**			
Mild to mod. flares	11 (6.01)	9 (7.03)	19 (8.92)
Severe flare	10 (5.46)	10 (7.81)	21 (9.86)
Total flares (mild to mod. + severe)	21 (11.48)	19 (14.84)	40 (17.84) (*p* = 0.060) ^b^
**Organ flares**			
Mucocutaneous system (alopecia, skin rash, vasculitis rash)	4 (2.19)	9 (7.03)	10 (4.69)
Hematological system (AIHA, leukopenia, thrombocytopenia)	4 (2.19)	2 (1.56)	4 (1.88)
Musculoskeletal system (myositis, arthritis)	1 (0.55)	0	3 (1.41)
Renal system	11 (4.37)	7 (5.57)	19 (8.92)
Nervous system (CNS and peripheral)	0	1 (0.78)	1 (0.47)
Gastrointestinal system	0	0	1 (0.47)
Constitutional symptoms	1 (0.55)	0	2 (0.94)
**IR (95% CI) of flares (events/100 person-months)**	8.88 (5.19–12.57)	6.55 (3.85–9.25)	7.12 (4.76–9.48)

Data are expressed as mean ± SD, median [p25–p75], or n (%); IR (95% CI) = incidence rate (95% confidence interval). * = last observation day prior to the next vaccination dose or study censor. Only significant *p*-values are shown. ^a^ = *p*-value of mSLEDAI-2K score between mRNA vaccine vs. adenovirus-vectored vaccine, ^b^ = *p*-value of total flares between mRNA vaccine and inactivated virus vaccine. AIHA = autoimmune hemolytic anemia, CNS = central nervous system, mSLEDAI-2K = modified Systemic Lupus Erythematosus Disease Activity Index-2000. mSLEDAI-K: analyzed by repeated mixed models; flares: analyzed by Poisson regression; incidence rate: analyzed by Poisson regression.

**Table 4:** Characteristics of patients compared between those with and without flares.

Baseline Characteristics	Cases with Flares (n = 62)	Cases Without Flares (n = 139)	*p*-Value
Female	58 (93.55)	133 (95.68)	0.502
Age in years	37.26 ± 10.64	43.21 ± 13.54	0.001
SLE disease duration in years	13.08 ± 8.47 11.43 [7.03–17.12]	14.20 ± 8.61 13.68 [6.88–21.73]	0.294
**Co** **-morbidity ** *			
Hypertension	20 (32.26)	45 (32.37)	0.987
Dyslipidemia	21 (33.87)	44 (31.65)	0.756
Thyroid disease	4 (6.45)	11 (7.91)	0.716
Diabetes mellitus	3 (4.84)	10 (7.19)	0.758
Previous malignancy	2 (3.23)	2 (1.44)	0.589
Hepatitis B or C infection	2 (3.23)	2 (1.44)	0.589
Others ^#^	0	4 (2.88)	0.314
**Current active organ involvement** **^†^**			
Renal system	30 (48.39)	33 (23.74)	0.001
Mucocutaneous system	17 (27.42)	8 (5.76)	<0.001
Musculoskeletal system	0	5 (3.60)	0.326
Hematological system	3 (4.84)	2 (1.44)	0.172
Gastrointestinal system	2 (3.23)	0	0.094
Neurological system	0	1 (0.72)	1.000
**mSLEDAI** **-2K score**			
6 months prior to 1st vaccine dose	3.72 ± 3.68 4 [0–4]	1.71 ± 2.73 0 [0–4]	<0.001
3 months prior to 1st vaccine dose	3.62 ± 4.15 2 [0–4]	155 ± 2.67 0 [0–4]	<0.001
1st vaccine dose (D1)	3.56 ± 4.14 2 [0–4]	1.50 ± 2.64 0 [0–4]	<0.001
Number of 1997 ACR classification criteria	5.92 ± 1.23	5.41 ± 1.22	0.007
Number of 2012 SLICC classification criteria	7.73 ± 1.84	6.75 ± 1.78	<0.001
SDI score	0.85 ± 1.30 0 [0–2]	0.93 ± 1.19 1 [0–2]	0.436
**Baseline medication ^†^**			
Prednisolone	56 (90.32)	118 (84.89)	0.297
Dose (mg/day)	7.63 ± 6.31 5 [5–10]	7.36 ± 7.10 5 [5–10]	0.547
Hydroxychloroquine	21 (33.87)	46 (33.09)	0.914
Dose (mg/day)	157.14 ± 57.63 200 [100–200]	146.85 ± 74.43 200 [100–200]	0.577
Immunosuppressive drug	36 (58.06)	70 (50.36)	0.312
Mycophenolate mofetil	27 (43.55)	54 (38.85)	0.530
Dose (mg/day)	1286.11 ± 748.95 1000 [500–2000]	1513.89 ± 671.90 2000 [1000–2000]	0.163
Cyclosporine	11 (17.74)	10 (7.19)	0.024
Dose (mg/day)	118.18 ± 40.45 100 [100,100]	150.00 ± 52.70 150 [100–200]	0.135
Azathioprine	4 (6.45)	10 (7.19)	0.849
Dose (mg/day)	50.00 ± 0.00 50 [50–50]	52.50 ± 27.51 50 [25–50]	0.740
Methotrexate	1 (1.61)	5 (3.60)	0.668
Dose (mg/week)	10.00 ± 0.00 10 [10–10]	11.00 ± 4.18 10 [10–15]	0.752
Cyclophosphamide	2 (3.23)	4 (2.88)	1.000
Dose (mg/month)	1250 ± 353.55 1250 [1000–1500]	925.00 ± 537.74 1000 [600–1250]	0.453
Tacrolimus	0	2 (1.44)	1.000
Dose (mg/day)		1.25 ± 1.06 1.25 [0.5–2]	

Data are expressed as mean ± SD, median [p25–p75], or n (%), n/N = number of positive tests/number tested. * = one patient might have more than one co-morbid disease, # others = major depression in two patients, cerebrovascular disease in two patients, end-stage renal disease in one patient, and congestive heart failure in one patient, ^†^ = at the time of the 1st vaccine dose. ACR = American College of Rheumatology, mSLEDAI-2K = modified Systemic Lupus Erythematosus Disease Activity Index-2000, SDI = Systemic Lupus International Collaborating Clinics/American College of Rheumatology Damage Index, SLICC = Systemic Lupus Erythematosus International Collaboration Clinics.

**Table 5:** Predicting factors for flares after vaccination.

Baseline Characteristics	Total	Flare (%)	Univariable			Multivariable		
			HR	95%CI	*p*-Value	HR	95%CI	*p*-Value
Female	191	58 (30.37)	0.94	0.34–2.60	0.902			
Age in years	41.37 ± 12.99	37.26 ± 10.64	0.97	0.95–0.99	0.004	0.98	0.96–1.00	0.089
SLE disease duration in years	13.86 ± 8.56	13.08 ± 8.47	0.98	0.95–1.01	0.323			
**Co** **-morbidity ** *								
Hypertension	65	20 (30.77)	1.03	0.61–1.77	0.900			
Dyslipidemia	65	21 (32.31)	1.20	0.70–2.04	0.510			
Thyroid disease	15	4 (26.67)	0.78	0.28–2.15	0.627			
Diabetes mellitus	13	3 (23.08)	0.55	0.17–1.76	0.312			
Previous malignancy	4	2 (50.00)	2.11	0.51–1.52	0.302			
Hepatitis B or C infection	4	2 (50.00)	3.71	0.88–15.59	0.073	3.53	0.83–15.04	0.089
**Current active organ involvement ** **^#^**								
Renal system	63	30 (47.62)	2.21	1.33–3.65	0.002	2.57	1.47–4.50	0.001
Mucocutaneous system	25	17 (68.00)	2.36	1.35–4.14	0.003	2.45	1.31–4.60	0.005
Musculoskeletal system	5	0						
Hematological system	5	3 (60.00)	1.89	0.59–6.07	0.286			
Gastrointestinal system	2	2 (100.00)	4.92	1.19–20.38	0.028	4.16	0.90–19.21	0.068
Neurological system	1	0						
mSLEDAI-2K score at 1st vaccine dose (D1)	2.13 ± 3.31	3.56 ± 4.14	1.14	1.07–1.21	<0.001	1.13	1.05–1.20	0.001 ^†^
Number of 1997 ACR classification criteria	5.57 ± 1.24	5.92 ± 1.23	1.28	1.05–1.55	0.014	1.22	1.00–1.50	0.053
SDI score	0.91 ± 1.22	0.85 ± 1.30	1.00	0.81–1.24	0.984			
**Baseline medication ^#^**								
Prednisolone	174	56 (32.18)	1.89	0.81–4.39	0.141			
Hydroxychloroquine	67	21 (31.34)	0.93	0.55–1.57	0.784			
Immunosuppressive drug	106	36 (33.96)	1.57	0.94–2.62	0.087			
Mycophenolate mofetil	81	27 (33.33)	1.22	0.74–2.03	0.437			
Cyclosporine	21	11 (52.38)	1.58	0.82–3.04	0.170			
Azathioprine	14	4 (28.57)	1.25	0.45–3.45	0.671			
Methotrexate	6	1 (16.67)	1.36	0.19–9.94	0.764			
Cyclophosphamide	6	2 (33.33)	2.49	0.60–10.31	0.208			
Tacrolimus	2	0						

* = one patient might have more than one co-morbid disease, ^#^ = at time of the 1st vaccine dose, ^†^ = *p*-value in multivariable analysis using mSLEDAI-2K score for renal, mucocutaneous, and gastrointestinal involvement. ACR = American College of Rheumatology, HR = hazard ratio, mSLEDAI-2K = modified Systemic Lupus Erythematosus Disease Activity Index-2000, SDI = Systemic Lupus International Collaborating Clinics/American College of Rheumatology Damage Index, SLICC = Systemic Lupus Erythematosus International Collaboration Clinics. Analyzed by Cox regression.

**Table 6:** Flares according to mSLEDAI-2K score.

	Flare: n/N (%)		*p*-Value
	mSLEDAI-2K Score = 0–5	mSLEDAI-2K Score ≥ 6	
**By dose of vaccine**			
1st dose (N = 201)	11/178 (6.18)	7/23 (30.43)	<0.001
2nd dose (N = 199)	33/174 (18.97)	7/25 (28.00)	0.368
3rd dos (N = 124)	18/113 (15.93)	4/11 (36.36)	0.081
**By type of vaccine**			
Inactivated virus (N = 183)	13/154 (8.44)	8/29 (27.59)	0.008
Adenovirus-vectored (N = 128)	16/113 (14.16)	3/15 (20.00)	0.621
mRNA (N = 123)	33/198 (16.67)	7/15 (46.67)	0.001

mSLEDAI-2K = modified Systemic Lupus Erythematosus Disease Activity Index-2000.

## Data Availability

The datasets used and/or analyzed during this study are available from the corresponding author upon reasonable request.

## Supplementary Materials

The following supporting information can be downloaded at https://www.mdpi.com/article/10.3390/vaccines12121399/s1: Table S1: Disease activity, flares, and factors associated with flare in SLE patients receiving COVID-19 vaccines (selected series).
