# Supplementary material for: Flares and Predicting Factors of Flares in Patients with Systemic Lupus Erythematosus Associated with Different Doses and Types of COVID-19 Vaccines
Source: Vaccines (Basel). 2024 Dec 12;12(12):1399. doi: 10.3390/vaccines12121399 (PMC11728829; doi:10.3390/vaccines12121399)
Supplement: Supplementary file 1 [file vaccines-12-01399-s001.zip › vaccines-3308054-supplementary.pdf]

Supplementary Table S1. Disease activity, flares, and factors associated with flare in SLE patients receiving COVID-19 vaccines (selected series)

Author, year	Country	type of study	No. of center	No. of SLE patient	Type of vaccine	Dose of vaccine	Assessment time	Disease activity	Flare definition/ instrument	Baseline SLE disease activity (SLEDAI score)	Changes in disease activity	Flare %	Severity of flares	Risk or factors associated with flares
**Single type of vaccine**														
Barbhaiya M, et al. 2022	USA	Cross.	Single	136	mRNA	≥1	2 weeks after vaccination	NA	NA	NA	NA	11/136 (8.01%) patients with 12 flares. 1st dose 8/12 (66.67%), 2nd dose 4/12 (33.33%)	1st dose: mild in 87.5%, 2nd dose: moderate in 75%. Most flares were related to MS and skin systems	
Yoshida T, et al. 2022	Japan	Retro.	Single	74	mRNA	2	30, 60, 90 days after 2nd dose	SLEDAI-2K	SELENA-SLEDAI	SLEDAI 1-5 in 43.24%, 6-10 in 35.14%, and ≥11 in 21.62%,	no changes in SLEDAI score	15/74 (20.27%) at 30 days, 19/74 (25.68%) at 60 days, and 12/74 (16.21%) at 90 days.	M&M: 15/15 (100%) at 30 days, 18/19 (94.74%) at 60 days, and 11/12 (91.67%) at 90 days. Severe: 0% at 30 days. 5.26% at 60 days, and 8.33% at 90 days	
Mormile I, et al. 2022	Italy	Pro.	single	41	mRNA	2	3 weeks after 1st dose, and 4 weeks after 2nd dose.	SELENA-SLEDAI	SELENA-SLEDAI	6±6.67 (SLEDAI <6 in 56.25%)	No changes in SLEDAI score	No flares		
Izmirly PM, et al. 2022	USA	Pro.	Single	90	mRNA	2	4 months after 1st dose	hybrid SELENA-SLEDAI	SELENA-SLEDAI	3.15 ±3.77	no changes in SLEDAI-score	9/79 (11.39%)	M&M: 8/9 (88.89%), severe: 1/9 (11.11%)	
Delkash P, et al. 2023	Iran	Cross.	Single	72	Inact.V.	≥1	12 weeks after last dose	SLEDAI-2K	SLEDAI-2K	0 (all in remission)	Median (IQR) 4 (4-4)	17/72(19.77%). 1st dose 6/14 (42.86%), 2nd dose 8/14 (57.12%)	arthritis in 9 (64.29%), skin in 3 (21.43%)	
Ma M, et al. 2023	Singapore	Retro.	Multi*	641/4,267 (13.85%) of the study	mRNA	≥1	12 weeks after 1st dose	NA	New symptoms requiring treatment with corticosteroids, immunosuppressive drugs, or hospitalization	NA	NA	51/641 (7.96%) within 3 months, 21/641 (3.28%) after 3 months, 73/641 (11.39%) improved		
Gonzalez-Melendez A, et al. 2023	Puerto Rico	Retro.	Single	247	mRNA	≥1	Up to 12 months after 1st dose	SLEDAI	Increase in disease activity requiring treatment	NA	No changes in SLEDAI score among those with flares	14/247 (5.67%). 1st dose 5/14 (35.71%), 2nd dose 9/14 (64.28%)	10/14 (71.43%) had major organ involvement (renal in 7, and one each with CNS, lung, and liver)	Higher baseline SLEDAI score, with a higher proportion of photosensitivity, mouth ulcers, anti-Ro antibodies, past exposure to IVMP therapy, and current corticosteroid use in the flare group
Kikuchi J, et al. 2024	Japan	Pro.	Single	90	mRNA	2	next visit after 2nd dose	SLEDAI-2K	SLELENA-SLEDAI	Median (IQR) 2.0 (0-2)	Increased SLEDAI score after 2nd dose	13/90 (14.44%)	M&M: 9/13 (69.23), severe: 4/13 (30.76%)	Higher SLEDAI score, higher anti-dsDNA antibody levels, and higher proportion of rash, and use of azathioprine,

														prior to 1st vaccine dose were observed among patients with flares. SLEDAI score and anti-dsDNA antibody titers were associated with flares
Zavala-Flores E, et al. 2022	Peru	Pro.	single	100	mRNA	≥1	10 days after vaccination	NA	Increase in disease activity or laboratory in one organ			20/100 (20.00%) patients with 27 flares. 1st dose 9/100 (9.00%), 2nd dose 18/90 (20.00%)	Mainly arthritis and skin.	Renal involvement and HCQ use decreased the risk of flares, but azathioprine use prior to immunization increased it
Fan Y, et al. 2022	China	Cross.#	Single	614	Inact.V.	≥1	NA	NA	patients reported flares	NA	NA	65/614 (10.58%)	19/614 (3.09%) requiring treatment escalation	
**Mixed type of vaccine**														
Mok CC, et al. 2022	Hong Kong	Retro.	single	449	Inact.V. 38.5%, mRNA 61.5%	2	6 weeks after 2nd dose	Mostly disease quiescence	modified SELENA-SLEDAI	NA		37/449 (8.24%)	M&M: 34/37 (91.89%) [renal in 16 (43.24%), MC in 13 (35.14%), arthritis in 8 (31.62%), serositis in 3 (8.11%) and thrombocytopenia in 2 (5.41%)]. Severe: 3/37 (8.11%) [renal in 2 (5.41%), GI in 1 (2.70%)]	Patients at younger age, receiving glucocorticoid treatment in the previous 3 months, having history of discoid lesions, arthritis, and positive anti-Sm/nRNP antibodies, and currently having active lupus serology before vaccination were more likely to have flares. Active lupus serology prior to vaccination, history of arthritis and discoid lesions were associated with flares
So H. et al. 2022	Hong Kong	Pro.	Single	65	Inact.V. 42%, mRNA 58%	2	4 weeks after 2nd dose	SELENA-SLEDAI-2K	SELENA-SLEDAI-2K	2.9 ± 2.0	No changes in SLEDAI-2K score	No flares	More patients had numerically reduced SLEDA-2K score, anti-dsDNA level and proteinuria	
Gerosa M, et al. 2022	Italy	Retro.	Multi.	452	Ade.V.= 1.55%, mRNA =98.45%	2	First FU visit after vaccine date	BILAG	SLEDAI-2K or BILAG (new BILAG A or B)	Remission or in LLDAS in 90%	No changes in proportion of BILAG domain	19/542 (3.51%)	MS in 6 (31.57%), renal in 4 (21.05%), CP in 3 (15.79%), hematological in 3 (15.79%), MC in 2 (10.53%)	
Assawasaksakul T, et al. 2022	Thailand	Pro.	Single	64 of 94 in the study	Inact.V. 24.47%, Ade.V. 45.74%, and mRNA 29.79%	2	4 weeks after 2nd dose	SLEDAI	SELENA-SLEDAI	2.11-3.12 (according to vaccine type)	No changes in SLEDAI	No flares		
Larsen E, et al. 2023	Denmark	Pro.	Single	123 (2 doses), 112 (3 doses)	Ade.V.6.5%., mRNA 93.5%	3	4-8 weeks after 2nd and 3rd dose	SLAQ by patients, SLEDAI-2K by physicians.	SLEDAI-2K or BILAG (BILAG A or B)	SLEDAI <4 in 60.2%	No changes in SLAQ and SLEDAI scores at baseline	No flares		

											and after 2nd dose			
Felten R, et al. 2021	France	Cross.#	Multi.**	696	Inact.V. 22.41%, Ade.V. 10.49%, and mRNA 65.51%	≥1	NA	NA	Patients reported medically confirmed flares	NA	NA	21/696 = 3.01%	MS in 19/21 (90.48%). Hospitalized in 4 (19.08%)	Flare in the past year was associated with flares after vaccination
Rider G, et al. 2022	USA	Retro.#	Multi.***	791/5,619 (14.07%) of the survey	NA	≥1	NA	NA	Patients reported change of treatment	NA	NA	53/791 = 6.70%,		
Present study	Thailand	Retro.	Single	201	Inact.V. 34.92%, Ade.V.24.43%, mRNA 40.65	≥1	90 days after the last dose or study censor	mSLEDAI-2K	mSELENA-SLEDAI-2K	2.13 ± 3.31	No changes in mSLEDAI-2K score	1st dose in 18/201 (8.96%), 2nd dose in 40/199 (20.10%), 3rd dose in 22/124 (17.74%)	1st dose: M&M: 6/18 (33.33%), severe: 12/18 (66.67%). 2nd dose: M&M: 23/40 (57.50%), severe: 17/40 (42.5%), 3rd dose: M&M: 10/22 (45.45%), severe: 12/22 (54.54%)	Patients at younger age at onset, with current renal and MC involvement, higher mSLEDAI-2K scores, and more mean cumulative number of 1997 ACR or 2012 SLICC criteria were more likely to have flares. Renal and MC involvement or higher mSLEDAI-2K scores predicted flares

* = Coronavirus National Vaccine registry for ImmuNe diseases SINGapore (CONIN-SING), ** = international vaccination against COVID in systemic lupus (VACOLUP), *** = COVID-19 Global Rheumatology Alliance (GRA), # = Web-based survey.
Pro. = prospective, Retro. = retrospective, Cross. = Cross-sectional, multi. = multicenter.
Inact.V = Inactivated vaccine, Ade.V. = Adenovirus-vectored vaccine, mRNA = mRNA vaccine.
Blank = no information provided, CNS = central nervous system, CP = cardiopulmonary, GI = gastrointestinal, IQR = interquartile range, IVMP = intravenous methylprednisolone, M&M = mild to moderate, MC = mucocutaneous, MS = musculoskeletal.
